# Next-generation sequencing of tyrosine kinase inhibitor-resistant non-small-cell lung cancers in patients harboring epidermal growth factor-activating mutations

**DOI:** 10.1186/s12885-015-1925-2

**Published:** 2015-11-16

**Authors:** Katsuhiro Masago, Shiro Fujita, Miho Muraki, Akito Hata, Chiyuki Okuda, Kyoko Otsuka, Reiko Kaji, Jumpei Takeshita, Ryoji Kato, Nobuyuki Katakami, Yukio Hirata

**Affiliations:** 1 Division of Integrated Oncology, Institute of Biomedical Research and Innovation, 2-2 Minatojima-minamimachi, Cyuo-ku, Kobe City, Hyogo 650-0047 Japan; 2 Thermo Fisher Scientific, Tokyo, Japan

**Keywords:** Acquired resistance, Epidermal growth factor, Next-generation sequencing, Tyrosine kinase inhibitor

## Abstract

**Background:**

The aim of this study was to detect the epidermal growth factor receptor (EGFR)-activating mutations and other oncogene alterations in patients with non-small-cell lung cancers (NSCLC) who experienced a treatment failure in response to EGFR-tyrosine kinase inhibitors (TKIs) with a next generation sequencer.

**Methods:**

Fifteen patients with advanced NSCLC previously treated with EGFR-TKIs were examined between August 2005 and October 2014. For each case, new biopsies were performed, followed by DNA sequencing on an Ion Torrent Personal Genome Machine (PGM) system using the Ion AmpliSeq Cancer Hotspot Panel version 2.

**Results:**

All 15 patients were diagnosed with NSCLC harboring *EGFR*-activating mutations (seven cases of exon 19 deletion, seven cases of L858R in exon 21, and one case of L861Q in exon 21). Of the 15 cases, acquired T790M resistance mutations were detected in 9 (60.0 %) patients. In addition, other mutations were identified outside of *EGFR*, including 13 cases (86.7 %) exhibiting *TP53* P72R mutations, 5 cases (33.3 %) of *KDR* Q472H, and 2 cases (13.3 %) of *KIT* M541L.

**Conclusions:**

Here, we showed that next-generation sequencing (NGS) is able to detect *EGFR* T790M mutations in cases not readily diagnosed by other conventional methods. Significant differences in the degree of *EGFR* T790M and other *EGFR*-activating mutations may be indicative of the heterogeneity of disease phenotype evident within these patients. The co-existence of known oncogenic mutations within each of these patients may play a role in acquired EGFR-TKIs resistance, suggesting the need for alternative treatment strategies, with PCR-based NGS playing an important role in disease diagnosis.

## Background

Recent advances in biomedical research have provided a greater understanding of the molecular basis of disease, with significant implications for therapeutic intervention. Somatic mutations, such as epidermal growth factor receptor (EGFR) mutations and anaplastic lymphoma kinase (ALK) gene rearrangements, play a significant role in the pathogenesis of non-small-cell lung cancer (NSCLC), with treatment decisions often based upon the outcome of these genetic tests [1–5].

Both *EGFR* and *ALK* function as a receptor tyrosine kinase, which are readily inhibited by a series of tyrosine kinase inhibitors (TKI), including gefitinib [6], erlotinib [7], and crizotinib [2]. Despite the initial treatment efficacy of these TKIs for the treatment of NSCLC, acquired resistance was found to develop in almost all cases. The well-known mechanism of acquired EGFR-TKIs resistance include second site mutations within the *EGFR* kinase domain [8, 9], up-regulation of alternative signaling pathways, such as *MET* [10], histologic transformation, epithelial to mesenchymal transition, and small cell transformation [11]. Although many resistance mechanisms have been clarified, the *EGFR* kinase domain mutation T790M in exon 20 accounts for nearly half of all acquired resistance, making testing for this mutation a key factor in determining following treatment strategies in the era of second- and third-generation EGFR-TKIs [12, 13].

The recent development of next-generation sequencing (NGS) as a diagnostic tool in the clinical setting has enabled us to determine rapid, targeted sequencing of tumors for causative mutations. When combined with various selective capture approaches, NGS has allowed for the efficient simultaneous genetic analysis of a large number of candidate genes. Here, we applied a polymerase chain reaction (PCR) based NGS in determining oncogene alternations in the state of disease progression.

PCR based next-generation sequencing is an outstanding tool to provide a comprehensive genomic diagnosis in patients with recurrent NSCLC [14]. The primary aim of this study was to evaluate *EFGR* T790M secondary mutations, along with other oncogenic alterations, in NSCLC patients previously diagnosed with *EGFR* activating mutations who experienced disease recurrence after treatment with first-generation EGFR-TKIs.

## Methods

### Patients and treatment regimens

Fifteen patients with NSCLC previously treated with EGFR-TKIs were examined between August 2005 and October 2014 at the Institute of Biomedical Research and Innovation in Kobe City, Japan. Patients were treated with either of erlotinib or gefitinib daily, at initial daily doses of 150 (erlotinib) and 250 (gefitinib) mg/day. Standard Response Evaluation Criteria in Solid Tumors (RECIST 1.0) was used to evaluate treatment response. Toxicities were graded according to the Common Terminology Criteria for Adverse Events (CTCAE) version 4.0. We obtained written informed consents from all the participants. This study was approved by the Research Ethics Committee of the Institute of Biomedical Research and Innovation.

### EGFR mutational analysis

A quantity of cancer cells sufficient for a pathologic diagnosis (i.e., several hundred cells) were obtained from formalin-fixed paraffin-embedded (FFPE) biopsy specimens by manual micro-dissection. Similar biopsy specimens were used to analyze *EGFR* somatic mutations in exons 18–21 [15, 16].

### MET gene amplification

For each patient, DNA was extracted, and the concentration measured using a Nanodrop ND-1000 spectrophotometer (Nanodrop Technologies, Rockland, DE). *MET* copy number gains (CNG) analysis was performed using the One-Step Real Time PCR System (Thermo Fisher Scientific, Foster City, CA) under the following conditions: one cycle of 95 °C for 10 min followed by 40 cycles of 95 °C for 15 s and 60 °C for 1 min. The qPCR reaction mixture contained 10 μL of 2X TaqMan genotyping master mix, 1 μL of the TaqMan copy number target assay, 1 μL of the TaqMan copy number reference assay (RNase P, which is known to exist only in two copies in a diploid genome), 4 μL of nuclease-free water, and 4 μL of DNA (diluted to a concentration of 5 ng/μL). Each sample was run in a minimum of four replicates. Amplification results were then analyzed using the CopyCaller Software (Thermo Fisher Scientific) for post-PCR data analysis. To accurately detect *MET* CNG, we analyzed the previous reported region of *MET* [17], a region spanning the intron 20–exon 21 boundary (TaqMan copy number assay Hs02884964_cn).

### Ion torrent PGM library preparation and sequencing

An Ion Torrent adapter-ligated library was generated using an Ion AmpliSeq Library Kit 2.0 according to the manufacturer’s protocol (Thermo Fisher Scientific, Rev. 5; MAN0006735). Briefly, 50 ng of pooled amplicons and the Ion AmpliSeq Cancer Hotspot Panel version 2 (Thermo Fisher Scientific) were end-repaired, and Ion Torrent adapters P1 and A were ligated using DNA ligase. Following AMPure bead (Beckman Coulter, Brea, CA, USA) purification, the concentration and size of the library were determined using the Life Technologies StepOne system (Thermo Fisher Scientific) and Ion Library TaqMan quantitation assay kit (Thermo Fisher Scientific). Sample emulsion PCR, emulsion breaking, and enrichment were performed using the Ion PGM IC 200 Kit (Thermo Fisher Scientific), according to the manufacturer’s instructions. Briefly, an input concentration of one DNA template copy/Ion Sphere Particle (ISP) was added to the emulsion PCR master mix, and the emulsion was generated using the Ion Chef (Thermo Fisher Scientific). Next, ISPs were recovered and template-positive ISPs enriched using Dynabeads MyOne Streptavidin C1 beads (Thermo Fisher Scientific). Sequencing was undertaken using 314 BC chips on the Ion Torrent PGM for 65 cycles using barcoded samples. The totally turnaround time from library preparation to the end of sequencing is about 2 days.

### Variant calling

After sequencing, data were processed using the Ion Torrent platform-specific pipeline software Torrent Suite to generate sequence reads, trim adapter sequences, and remove poor signal-profile reads. Initial variant calling was generated using Torrent Suite Software v4.0 using the variant caller plug-in. To eliminate erroneous base calling, three filtering steps were used. The first filter was set at an average total coverage depth of >100, variant coverage of >20, and *P* values <0.01. The second filter was employed by visually examining mutations using the Integrative Genomics Viewer (http//www.broadinstitute.org/igv) or CLC Genomics Workbench version 7.04 (Qiagen) software. Finally, possible strand-specific errors, such as mutation only detected in only the plus or minus strand were removed.

## Results

A summary of patient characteristics can be found in Table 1. All patients were Japanese, consisting of 10 females (76.7 %) and 5 males (33.3 %). Nine patients (60.0 %) were never smokers, and the remaining six patients (40.0 %) were former smokers. All patients had stage IV adenocarcinoma, as defined based upon TNM classification criteria (7th edition) [18]. Eight patients received erlotinib, and four patients were treated with gefitinib. The remaining patient was treated first with gefitinib, then switched to erlotinib. The median duration of EGFR-TKI therapy was 510 days (range: 122–1912 days; Table 1).

**Table 1:** Patient characteristics

Patient characteristics	(%)
Age (years)	
Range	54–79
Gender	
Male	5 (33.3)
Female	10 (76.7)
Smoking status	
Non-smoker	9 (60.0)
Former Smoker	6 (40.0)
Stage	
IV	14 (93.4)
rIV^a^	1 (6.6)
1^st^ line	5 (33.3)
2^nd^ line	7 (46.7)
3^rd^ line	2 (13.4)
Subsequent therapy	1 (6.6)

^a^*rIV* recurrent stage IV

*EGFR* sequence variations are listed in Table 2. All patients were diagnosed with adenocarcinomas harboring *EGFR* activating mutations (seven cases of exon 19 deletion, seven cases of L858R in exon 21, and one case of L861Q in exon 21). Of the 15 cases, acquired *EGFR* T790M resistance mutations in exon 20 were detected in 9 (60.0 %) patients. Of particular interest were cases 7, 8, and 10, in which T790M mutations were not detected by high-sensitivity conventional PCR-based methods, such as peptide nucleic acid-locked nucleic acid (PNA-LNA) PCR clamp [16], or Cycleave real-time PCR [15].

**Table 2:** Clinical characteristics and next-generation sequencing results

	Histology	*EGFR* Sequence Variants	Frequency (%)	Allele Call	Exon 20 T790M	Frequency (%)	Conversion to SCLC	Prior TKIs	Duration (days)
Case 1	Adenocarcinoma	Exon 19	44.3	Heterozygous	Yes	7.2	No	Erlotinib	681
		E746_T750 del							
Case 2	Adenocarcinoma	Exon 19	59.4	Heterozygous	No	-	No	Gefitinib	537
		E746_T751 del > A							
Case 3	Adenocarcinoma	Exon 21 L858R	46.1	Heterozygous	No	-	No	Gefitinib	195
		Exon 18 T725R	30.6	Heterozygous					
Case 4	Adenocarcinoma	Exon 21 L858R	23.3	Heterozygous	No	-	No	Erlotinib	217
		Exon 20 S768I	10.0	Heterozygous					
Case 5	Adenocarcinoma	Exon21 L858R	56.9	Heterozygous	No	-	No	Gefitinib	1105
		Exon 18 E709G	54.5	Heterozygous					
Case 6	Adenocarcinoma	Exon 19	97.2	Homozygous	Yes	21.8	No	Erlotinib	693
		E746_T750 del							
Case 7	Adenocarcinoma	Exon 21 L858R	13.8	Heterozygous	Yes	5.2	No	Erlotinib	537
Case 8	Squamous cell carcinoma	Exon 19	86.9	Heterozygous	Yes	7.3	No	Erlotinib	315
		E746_T750 del							
Case 9	Adenocarcinoma	Exon 19	65.3	Heterozygous	Yes	41.3	No	Erlotinib	1555
		E746_T750 del							
Case 10	Adenocarcinoma	Exon21 L858R	11.2	Heterozygous	Yes	4.8	No	Gefitinib	1912
Case 11	Adenocarcinoma	Exon 19	46.4	Heterozygous	Yes	11.0	No	Erlotinib	256
		E746_T750 del							
Case 12	Adenocarcinoma	Exon21 L858R	22.2	Heterozygous	No	-	No	Erlotinib	924
		Exon 21 G873R	10.8	Heterozygous					
Case 13	Adenocarcinoma	Exon 21 L861Q	59.9	Heterozygous	No	-	No	Gefitinib Erlotinib	1304 122
		Exon 20 P772S	10.2	Heterozygous					
		Exon19 L747S	11.8	Heterozygous					
		Exon2 A289V	12.3	Heterozygous					
Case 14	Adenocarcinoma	Exon 19	80.82	Heterozygous	Yes	14.8	No	Erlotinib	392
		E746_T750 del							
Case 15	Adenocarcinoma	Exon21 L858R	76.7	Heterozygous	Yes	10.3	No	Erlotinib	339

**Fig. 1:**
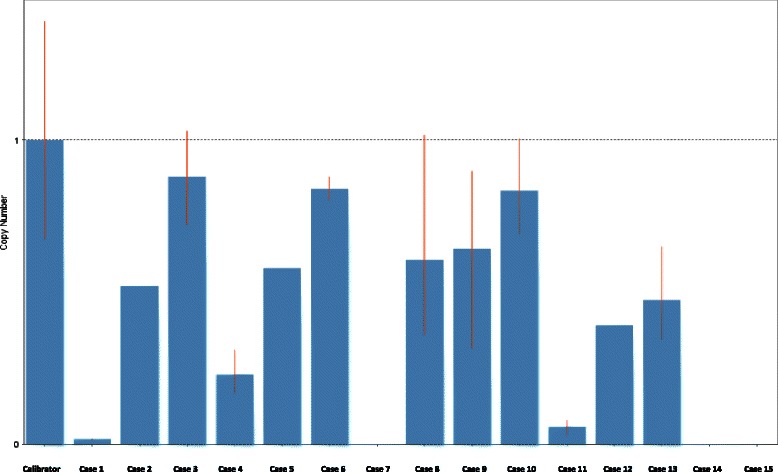
Quantitaive polymerase chain reaction (qPCR) *MET* copy number gain (CNG) analysis for 15 cases

In addition to T790M mutations, a large number of activating mutations were identified outside of *EGFR. MET* amplification, another common mutation associated with EGFR-TKI resistance, was not seen (Fig. 1), which is also confirmed by copy number analysis of NGS sequencing data (data not shown). Further screening of an additional 50 known oncogenes revealed a quite number of mutations in at least 32 genes (Table 3), including 13 cases (86.7 %) of *TP53* P72R mutations, 5cases (33.3 %) of *KDR* Q472H, and 2 cases (13.3 %) of *KIT* M541L. A full list of genes analyzed in this study is shown in Table 4.

**Table 3:** Coexisting somatic mutations resulting in amino-acid changes identified using the Ion AmpliSeq Hotspot Panel version 2

		Frequency (%)		Frequency (%)		Frequency (%)		Frequency (%)		Frequency (%)
Case 1	*KIT* M541L (COSM 28026)	70.9	*TP53* P72R	53.2	---	---	---	---	---	---
Case 2	*PTEN* L57W (COSM 5253)	21.2	---	---	---	---	---	---	---	---
Case 3	*TP53* P72R	57.0	*CTNNB1* D32N (COSM 5672)	34.5	*TP53* V73 del	29.1	*CDH1* Q346* (COSM 19524)	25.1	---	---
Case 4	*TP53* P72R	60.3	*TP53* R337C (COSM 11071)	18.0	---	---	---	---	---	---
Case 5	*TP53* P72R	46.9	*KDR* Q472H	46.9	*KIT* G534C	46.3	*APC* S1463fs	42.5	---	---
Case 6	*PDGFRA* P567Q	100	*TP53* V73W	72.6	*TP53* P151S	57.5	*KDR* Q472H	42.4	*ERBB4* C614Y	38.2
	*SMAD4* R189H	29.0	*PTEN* R233Q	18.5	*APC* D1591N	18.4	*HRAS* T64*	17.9	*AKT1* T21I	16.4
	*KIT* L647F	16.2	*SKT11* D352N	15.1	*PTEN* H123Y (COSM 5078)	7.3	*PTEN* R130Q (COSM 5033)	7.2	---	---
Case 7	*TP53* P72R	96.7	*SKT11* F345L	53.5	*SKT11* P281L	53.3	*KDR* Q472H	42.6	---	---
Case 8	*TP53* P72R	98.4	*KIT* M541L (COSM 28026)	59.8	*TP53* V154G (COSM 43903)	35.4	*KDR* Q472H	26.7	*SMAD4* G423R	14.7
	*ABL1* I347fs	11.1	*ERBB4* C759T	8.8	*FBXW7* M467I	8.0	*MLH1* A169V	8.0	*KDR* G1284R	7.9
	*APC* P1433L	6.7	*TP53* F338L	6.5	*SMO* P610S	6.4	*MET* D340A	5.8	*NOTCH1* V1575M	5.7
	*PTEN* A328E	5.6	*APC* G1374K (COSM 18737)	5.1	*MLH1* R148W	5.0	---	---	---	---
Case 9	*APC* E1464fs	59.2	*TP53* P72R	48.2	*BRAF* G442D	6.1	*MET* G1102D	5.5	*SMO* T223I	5.0
Case 10	*MET* N375K	55.7	*TP53* P72R	42.0	*CTNNB1* G34V	6.5	---	---	---	---
Case 11	*TP53* P72R	68.5	*PTEN* N329fs (COSM 4932)	39.5	*TP53* K132R (COSM 11582)	29.7	---	---	---	---
Case 12	*TP53* P72R	98.1	*KDR* Q472H	96.4	*TP53* V272fs	21.0	*RB1* I682T	12.6	*APC* P1433L	9.6
	*RET* E884V	9.1	*SMAD4* V354L	8.0	---	---	---	---	---	---
Case 13	*TP53* P72R	99.1	*CDKN2* G155S	51.6	*FLT3* W603*	45.2	*KRAS* E37K	33.3	*SMO* P641L	23.7
	*IDH1* L103M	20.0	*TP53* R267Q (COSM 43923)	18.8	*GNA11* D205N	16.2	*SMARCB1* P165S	14.0	*RB1* M761T	13.9
	*SMARCB1* V145L	12.4	*TP53* G245R (COSM 10957)	10.8	*NOTCH1* H1591T	10.7	*ERBB4* G240V	10.0	*KIT* S715N	9.9
	*FBXW7* R505H (COSM 25812)	9.8	*FBXW7* M498I	9.2	*MET* S186L	8.8	*IDH1* A111V	8.8	*JAC3* V133I	8.5
	*KIT* V825I (COSM 19110)	8.1	*TP53* G112S	6.5	*TP53* K132E (COSM 10813)	6.3	*HNF1A* A193V	6.3	*VHL* K171T	5.7
	*ALK* P1191A	5.6	*HNF1A* T204I	5.3	---	---	---	---	---	---
Case 14	*PTEN* H1047L	62.9	*FGFR3* R765S	7.2	*IDH1* P118L	5.7	---	---	---	---
Case 15	*TP53* P72R	100	*MET* A179M	5.1	---	---	---	---	---	---

**Table 4:** Target genes in the Ion AmpliSeq Hotspot Panel version 2

ABL1	EZH2	JAK3	PTEN
AKT1	FBXW7	IDH2	PTPN11
ALK	FGFR1	KDR	RB1
APC	FGFR2	KIT	RET
ATM	FGFR3	KRAS	SMAD4
BRAF	FLT3	MET	SMARCB1
CDH1	GNA11	MLH1	SMO
CDKN2A	GNAS	MPL	SRC
CSF1R	GNAQ	NOTCH1	STK11
CTNNB1	HNF1A	NPM1	TP53
EGFR	HRAS	NRAS	VHL
ERBB2	IDH1	PDGFRA	
ERBB4	JAK2	PIK3CA	

## Discussion

In this study we analyzed biopsy specimens of patients who underwent second biopsy after treatment failure with the first generation EGFR-TKIs. There was a significant difference between the frequency of *EGFR* T790M and other *EGFR*-activating mutations, with significant variability among cases (4.8–41.3 %). The existence of *EGFR* and other mutations within the same tumor sample identified by NGS highlights the importance of this type of analysis in guiding appropriate cancer therapy.

High-throughput sequencing was able to detect T790M mutation in a number of cases with the same accuracy of conventional highly sensitive conventional PCR methods, such as PNA-LNA PCR clamp [16] and Cycleave real-time PCR [15]. While high sensitivity and specificity of these methods is well established [19–27], the use of NGS provides important advantages with clarifying activating mutation rate in tumor sample as well as greater detection of rare mutations outside of target areas [28–31]. In addition, to emphasize the power of NGS in clinical practice, we should also try to develop its applications and usages such as challenging specimens or testing processes, such as peripheral blood in the future.

NGS is also able to overcome issue of germ-line DNA contamination, similar to that of new PCR methods, such as digital PCR [32]. This tolerance of germ-line DNA contamination allows for more streamlined sample preparation techniques, without need for time-consuming procedures such as macro- or micro-dissection. In this study, all samples were extracted from FFPE biopsy specimens, highlighting both versatility and potential use of NGS in clinical settings. Furthermore NGS is able to quantify gene mutations within a tumor sample. Due to the unpredictablity of PCR amplification and germ line DNA contamination, observed mutations does not always reflect the penetrance of a mutation within a sample. While most highly sensitive detection methods provide only categorical results such as positive and negative, our analysis was able to identify the degree of *EGFR* T790M and other *EGFR*-activating mutations within a sample that could not be explained by germ-line DNA contamination and/or PCR efficacy. These results are consistent with previous reports detailing T790M allelic frequency in terms of both intra-tumor heterogeneity in localized lung adenocarcinomas [33] and allelic imbalances [34]. Our analysis was able to identify the degree of *EGFR* T790M and other *EGFR*-activating mutations within a sample that could not be explained by germ-line DNA contamination and/or PCR efficacy. Future treatment with next-generation EGFR-TKIs targeting T790M is likely to be informed by such analyses, as patients should be treated based upon their *EGFR* acquired mutation [35].

In addition to *EGFR* mutations, we also evaluated another 50 oncogenes thought to have an important role in cancer pathogenesis (Table 4). A large number of mutations were identified in this analysis. However, how much extent these genes affect tumorigenicity, tumor progression, and resistance to EGFR-TKIs is difficult to assess, as some mutations may represent only passive alterations (passenger mutations). Although many of these mutations were identified in a single patient, a series of mutations including *TP53* P72R, *KDR* Q472R, and *KIT* M541L were detected in more than two cases, suggesting a role in disease progression.

*TP53* P72R was the most common mutation, detected in 13 of 15 cases (86.7 %). In human populations, *TP53* codon 72 is encoded by the nucleotide sequence CCC, which encodes proline, or CGC, which encodes arginine. While proline is the most common amino acid found at this residue, comparative sequence analyses have detected a high degree (>50 %) of *TP53*-R72 variants among certain populations [36]. The current understanding of *TP53* biology is that *TP53*-R72 is more effective at inducing apoptosis and protecting stressed cells from neoplastic development than the more common *TP53*-P72 [37]. However, it is not yet understood how these functional differences might translate between in vitro and in vivo settings [38, 39], making it difficult to assess the role of this sequence variant of EGFR-TKI resistance.

*KDR* (kinase insert domain receptor, also known as VEGFR2) is an important factor in tumor development and progression due to its pro-angiogenic effects [40]. *KDR* Q472H mutations were detected in 5 of 15 cases (33.3 %), making it the second most common gene variant observed outside of *EGFR*. In human populations, codon 472 of KDR is encoded by the nucleotide sequence CAA, which encodes glutamine, or CAT, which encodes histidine. The Q472H variant is thought to affect protein function due to increased phosphorylation after vascular endothelial growth factor (VEGF)-A stimulation, along with increased binding efficiency for VEGF-A165 [41]. The effect of Q472H on microvessel density is thought to occur as a result of increased phosphorylation of VEGFR2 [42]. Here, increased microvessel density may have contributed to EGFR-TKI resistance, suggesting that VEGFR2 inhibition may inhibition may become an important therapeutic option in patients with documented EGFR-TKI resistance.

V-Kit Hardy-Zuckerman 4 Feline Sarcoma Viral Oncogene Homolog (*KIT*) M541L substitutions were detected in 2 of 15 cases (13.3 %). c-KIT is one of the primary targets of imatinib, and mutations in *KIT* are predictive of the efficacy of the drug in gastrointestinal stromal tumors (GIST) [43]. Several case reports have suggested a potential role of the *KIT* M541L variant in the sensitivity of Imatinib for aggressive fibromatosis [44–46]. Furthermore, a wide array of in vitro analyses support a role for the L541 variant in tumorigenesis. FDC-P1 cells transfected with *KIT*-L541 showed an enhanced proliferative response, while *KIT*-L541 cells were more sensitive to imatinib than those expressing wild-type *KIT* [47]. Inokuchi, et al. observed a higher frequency of L541 variants among patients with chronic myelogenous leukemia (CML), which is consistent with increased tyrosine kinase activation and proliferative responses in *KIT*-L541 cells relative to wild-type controls [48]. From the view point of EGFR-TKI resistance, these data suggest a causative role for the *KIT* L541 variant in recurrence and drug resistance of NSCLC. Suppression of KIT with drugs like Imatinib may be a useful therapeutic choice in patients with *KIT*-variant tumors.

Five (cases 3, 4, 5, 12 and 13) out of six NSCLC patients that are negative for *EGFR*-T790M mutation harbored “compound mutations” (a rare *EGFR* mutation in combination with a more frequent activating mutation). On the other hand, all T790M-positive tumors (cases 1, 6, 7, 8, 9, 10 and 11) lack an additional rare mutation apart from the presence of a frequent inhibitor-sensitive *EGFR* mutation. Among these compound mutations (specifically rare mutations), tumors harboring S768I in exon 20 is known as resistant to EGFR-TKIs. On the contrary, tumors harboring point mutations in exon 18 and dual mutation of exon 19 deletion and S768I are reported to possible response to EGFR-TKIs. There have been limited data in other compounds mutations. So a role of these mutations in causing drug resistance in T790M-negative patients is uncertain and need to be evaluated [49].

This study has its limitations. The strongest limitations include a small sample size, and the retrospective nature of the study preventing the comparison of our findings to non-lesional or pre-treatment results. With this limitation of not having pre-treatment results, the role of activating mutations in additional oncogenes in TKI-resistance may be the primary cause for TKI resistance especially in the case of *KDR* Q472H mutations. A larger prospective study with strict enrollment criteria is definitely needed to overcome these limitations.

## Conclusion

In conclusion, our study showed that NGS could be useful to detect *EGFR* T790M variants in patients not otherwise found with other conventional PCR based methods. Furthermore, our results highlight the difference of the extent of EGFR T790M and other EGFR-activating mutations among tumor samples, which may indicate the heterogeneity of acquired mutations. Identification of additional sequence variations in potential oncogenes that may affect EGFR-TKI resistance would suggest a series of new therapeutic agents targeting on a patient’s underlying genetic profile.
